# A chromosome-level genome assembly of an early matured aromatic *Japonica* rice variety Qigeng10 to accelerate rice breeding for high grain quality in Northeast China

**DOI:** 10.3389/fpls.2023.1134308

**Published:** 2023-02-23

**Authors:** Shukun Jiang, Xijuan Zhang, Xianli Yang, Chuanzeng Liu, Lizhi Wang, Bo Ma, Yi Miao, Jifang Hu, Kefei Tan, Yuxian Wang, Hui Jiang, Junhe Wang

**Affiliations:** ^1^ Qiqihar Branch of Heilongjiang Academy of Agricultural Sciences, Qiqihar, China; ^2^ Heilongjiang Provincial Key Laboratory of Crop Physiology and Ecology in Cold Region, Heilongjiang Provincial Engineering Technology Research Center of Crop Cold Damage, Harbin, China; ^3^ Northeast Branch of National Salt-Alkali Tolerant Rice Technology Innovation Center, Harbin, China; ^4^ Crop Cultivation and Tillage Institute of Heilongjiang Academy of Agricultural Sciences, Harbin, China; ^5^ Keshan Branch of Heilongjiang Academy of Agricultural Sciences, Qiqihar, China

**Keywords:** aromatic *japonica* rice, genome assembly, early-matured, northern limit region, functional genes

## Abstract

Early-matured aromatic *japonica* rice from the Northeast is the most popular rice commodity in the Chinese market. The Qigeng10 (QG10) was one of the varieties with the largest planting area in this region in recent years. It was an early-matured *japonica* rice variety with a lot of superior traits such as semi-dwarf, lodging resistance, long grain, aromatic and good quality. Therefore, a high-quality assembly of Qigeng10 genome is critical and useful for japonica research and breeding. In this study, we produced a high-precision QG10 chromosome-level genome by using a combination of Nanopore and Hi-C platforms. Finally, we assembled the QG10 genome into 77 contigs with an N50 length of 11.80 Mb in 27 scaffolds with an N50 length of 30.55 Mb. The assembled genome size was 378.31Mb with 65 contigs and constituted approximately 99.59% of the 12 chromosomes. We identified a total of 1,080,819 SNPs and 682,392 InDels between QG10 and Nipponbare. We also annotated 57,599 genes by the Ab initio method, homology-based technique, and RNA-seq. Based on the assembled genome sequence, we detected the sequence variation in a total of 63 cloned genes involved in grain yield, grain size, disease tolerance, lodging resistance, fragrance, and many other important traits. Finally, we identified five elite alleles (*qTGW2^Nipponbare^*, *qTGW3^Nanyangzhan^*, *GW5^IR24^*, *GW6^Suyunuo^*, and *qGW8^Basmati385^*) controlling long grain size, four elite alleles (*COLD1^Nipponbare^*, *bZIP73^Nipponbare^*, *CTB4a^Kunmingxiaobaigu^*, and *CTB2^Kunmingxiaobaigu^*) controlling cold tolerance, three non-functional alleles (*DTH7^Kitaake^*, *Ghd7^Hejiang19^*, and *Hd1^Longgeng31^*) for early heading, two resistant alleles (*Pia^Akihikari^* and *Pid4^Digu^*) for rice blast, a resistant allele *STV11^Kasalath^* for rice stripe virus, an *NRT1.1B^IR24^* allele for higher nitrate absorption activity, an elite allele *SCM3^Chugoku117^* for stronger culms, and the typical aromatic gene *badh2-E2* for fragrance in QG10. These results not only help us to better elucidate the genetic mechanisms underlying excellent agronomic traits in QG10 but also have wide-ranging implications for genomics-assisted breeding in early-matured fragrant *japonica* rice.

## Introduction

Rice (*Oryza sativa* L.) is a safe and staple food source for more than half of the world’s population and serves as a model plant for cereal genetic studies (Gross and Zhao, 2014). Novo sequencing and genomic technologies have been widely applied in rice to promote the shift of breeding schemes from conventional field selection to genomic-assisted breeding (Gu et al., 2022). *O. sativa subsp. japonica*/*Geng* and *subsp. Indica*/*Xian* are the two major subspecies of cultivated rice (Zhang et al., 2016a; Nie et al., 2017). The *japonica*/*Geng* rice planting area is 9.87 million ha, accounting for approximately 32.9 percent of the total rice planting area in China (Tang and Chen, 2021). Recently, the early-matured *japonica*/*Geng* rice is becoming more and more important, and its growing area was more than 4 million ha in Northeast China (Cui et al., 2022). Two genome draft sequences of the cultivated rice subspecies *japonica*/*Geng* Nipponbare and *Indica*/*Xian* 93-11 were released in 2002 (Goff et al., 2002; Yu et al., 2002). In 2005, the International Rice Genome Sequencing Project (IRGSP) published the first completed version of the Nipponbare sequence (International-Rice-Genome-Sequencing-Project, 2005). Over the last two decades, several pan-genomes including 66 rice genomes (Zhao et al., 2018b), 33 rice genomes (Qin et al., 2021), 111 rice genomes (Zhang et al., 2022a),251 rice genomes (Shang et al., 2022), and 12 *japonica* rice genome (Wang et al., 2023) were built including IR64, R498, Zhenshan 97, Minghui 63, Taichung Native 1, LTH, Kitaake, IR8, N22, Huajingxian74, HR12, Basmati 334, Dom Sufid, Huazhan and Tianfeng at the chromosome level, and Shennong265, DJ123, WR04-6, Suijing18, Koshihikari, Basmati, Kongyu-131, and Guangluai-4 at scaffold level have been assembled and released with unprecedented speed (Mahesh et al., 2016; Zhang et al., 2016b; Du et al., 2017; Li et al., 2017; Nie et al., 2017; Stein et al., 2018; Zhao et al., 2018b; Jain et al., 2019; Choi et al., 2020; Tanaka et al., 2020; Panibe et al., 2021; Li et al., 2021a; Yang et al., 2022; Zhang et al., 2022b). These assembled genome sequences will be helpful in pinpointing new causal variants that underlie complex agronomic traits and identifying many of the genome-specific loci that were absent from the Nipponbare reference genome. However, most of these varieties are *Indica*/*Xian* rice or landrace. Nevertheless, the genome of *japonica*/*Geng* differs significantly from that of *indica*/*Xian* (Nie et al., 2017). Since the release of the finished version genome of Nipponbare, only seven genomes at the scaffold level of early-matured *japonica*/*Geng* varieties in northern region of China including Shennong265, Liaogeng5, Yanfeng47, Suijing18, Longgeng31, Daohuaxiang2(Wuyoudao4), and Kongyu-131 were released (Nie et al., 2017; Li et al., 2018; Zhao et al., 2018b; Wang et al., 2023). Only Daohuaxiang2 and Suijing18 were belong to early-mature aromatic type. The public availability of *japonica*/*Geng* genomes at the chromosome level, especially for the early-mature aromatic type, remains largely blank (Nie et al., 2017). Moreover, a few genomes are not enough to represent the whole genomic content of the *japonica* rice. The novo assembled genomes of an early-mature aromatic variety would be advantageous for functional genomics and genome research. For example, if there is structural variation in the particular variety and the reference genome in the candidate region, the guiding role of the reference genome would be limited. So, there is still a need for *de novo* genome assembly for various purposes especially in early-mature aromatic rice breeding research.

Fragrant and long grain are key grain quality traits that directly influence the global market price of rice (Hui et al., 2022). The basmati rice and jasmine rice are the two most popular fragrant *indica* rice in the world. However, consumers from East Asia, including North China, Japan, and Korea tend to prefer *japonica* rice (Lu et al., 2022). So, the aromatic long grain rice from Northeast China, represented by Wuyoudao4 (WYD4), is the most famous rice in the Chinese market. WYD4 had a superior quality, but also had a number of defects, most importantly, poor lodging resistance, lack of cold tolerance and blast resistance, and late maturity (Gao et al., 2012). In 2019, Qiqihar Branch of Heilongjiang Academy of Agricultural Sciences developed Qigeng10 (QG10) to solved these defects of WYD4. The plant area of QG10 was 0.4 million ha in the recent three years. QG10 has been a major variety of early matured aromatic long grain rice in Northeast China. The construction of a high-quality chromosome-level genome of QG10 is very important for improving the efficiency of rice genetic mechanism studies for desirable agronomic traits, such as eating quality, cold tolerance, lodging tolerance and early maturity, as well as accelerating the process of high-quality rice breeding in cold region of northeast China by design (Li et al., 2021a). Here, we produced a high-precision QG10 chromosomal genome by performing whole-genome sequencing in the Nanopore platform (Lin et al., 2021), followed by the Hi-C-assisted assembly mount technology (Van Berkum et al., 2010). Our results provided several functionally important candidate alleles for the grain length, cold tolerance, early heading, disease resistance, lodging resistance, and nitrate-use of rice breeding in cold region of northeast China.

## Materials and methods

### Materials

**Figure 1:**
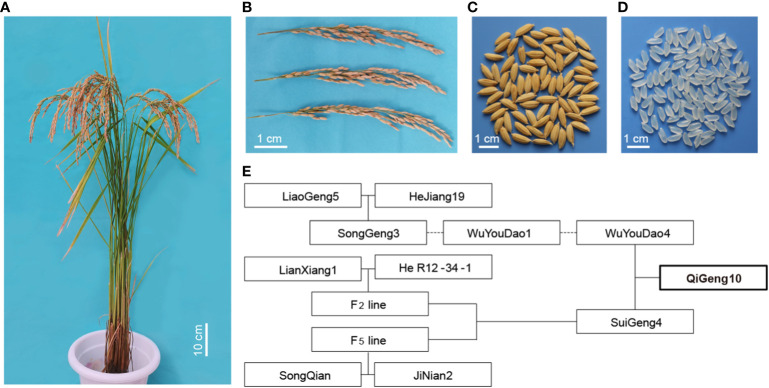
The of plant type, panicle phenotype, seeds phenotype, grains phenotype and pedigree of QG10. **(A)** The plant type of QG10; **(B)** The panicle morphology of QG 10; **(C)** The seed morphology of QG10; **(D)** The grain morphology of QG10; **(E)** The pedigree of QG10.

The early-matured aromatic long-grain *japonica* rice variety QG10, which was developed by our own group, was licensed for release in 2019 and is now widely planted in Heilongjiang province in Northeast China. It was a semi-dwarf rice variety with a lot of superior traits such as long panicle, long grain, aromatic, and good quality (**Figures 1A-D**). It was selected from the cross between two aromatic *japonica*/*Geng* rice Wuyoudao4 (WYD4) and Suigeng4 (SG4) (**Figure 1E**). The seedlings of QG10 were grown on the agricultural farm of the Qiqihar Branch of Heilongjiang Academy of Agricultural Sciences. Field management practices were performed according to the most commonly followed agricultural practices of local farmers. The leaves, stems, roots, and panicles at heading stages from plants grown in the experimental station were collected in liquid nitrogen for isolating RNA. The young leaves of a single young plant were used to isolate genomic DNA.

### Oxford Nanopare sequencing and genome assembly

The high molecular weight genomic DNA of QG10 was extracted from the 15-day-old leaf tissues following a modified CTAB method. Whole genome sequencing was done following the standard instructions of the Ligation Sequencing Kit (Nanopore, Oxford shire, UK). The quantified DNA was randomly sheared, and fragments of ∼20 kb were enriched and purified. Then, a 20-kb library was constructed and sequenced on the Nanopore PromethION platform according to the manufacturer’s protocols (Jiang et al., 2020).

*De novo* genome assembly of Nanopore sequence was performed as follow: The raw Nanopore reads were error-corrected and assembled using CANU (v1.7.1) (Koren et al., 2017), followed by Smartdenovo (https://github.com/ruanjue/smartdenovo) assembly, followed by three rounds of polishing with Racon (Vaser et al., 2017), followed by three rounds with Pilon v0.3.0 using the Illumina PCR-free paired-end reads (Walker et al., 2014). Genome completeness was also assessed using the algae dataset of BUSCO v2.0 (Simão et al., 2015).

### Hi-C library construction and sequencing

A Hi-C fragment library with a 300-700 bp insert size was constructed from the genomic DNA of the QG10. Briefly, adapter sequences of raw reads were trimmed and low-quality PE reads were removed for clean data. The library was sequenced on the Illumina HiSeq4000™ platform by Biomarker Technologies (Beijing, China). The clean Hi-C reads were first truncated at the putative Hi-C junctions and then the resulting trimmed reads were aligned to the assembly results with the software package bwa aligner (Li and Durbin, 2009). Only uniquely alignable pair reads whose mapping quality of more than 20 retained for further analysis. Invalid read pairs, including dangling-end and self-cycle, re-ligation, and dumped products, were filtered by the software package HiC-Pro v2.8.1 (Servant et al., 2015). The 96.25% of unique mapped read pairs were valid interaction pairs and were used for the correction of scaffolds and clustered, ordered, and orientated scaffolds onto chromosomes by the software package LACHESIS (Burton et al., 2013).

### Genome assembly and Hi-C scaffolding

Before chromosome assembly, we first performed a preassembly for error correction of scaffolds which required the splitting of scaffolds into segments of 50 kb on average. The Hi-C data were mapped to these segments using the software package BWA (version 0.7.10-r789) (Li and Durbin, 2009). The uniquely mapped data were retained to perform assembly by using the software package LACHESIS (Burton et al., 2013). Any two segments which showed an inconsistent connection with information from the raw scaffold were checked manually. These corrected scaffolds were then assembled with the software package LACHESIS. After this step, placement and orientation errors exhibiting obvious discrete chromatin interaction patterns were manually adjusted.

### Gene prediction and genome annotation

The RNA of QG10 were isolated from the mixed tissues (leaves, culms, roots, and panicles) following the manufacturer’s protocol (Wang et al., 2023). We then performed the sequencing on the Illumina HiSeq 2500 platform according to the manufacturer’s instructions. The repetitive sequence of the genome based on the principle of structure prediction and *de-novo* prediction was constructed with the software package LTR_FINDER v1.05 (Xu and Wang, 2007) and the software package RepeatScout v1.0.5 (Price et al., 2005). The PASTE Classifier was used to classify the database (Hoede et al., 2014). Then it was merged with the database of Repbase as the final repeat sequence database (Jurka et al., 2005). And then the software package RepeatMasker v4.0.6 was used to predict the repeat sequence of the QG10 genome based on the constructed repetitive sequence database (Tarailo‐Graovac and Chen, 2009). the software packages Genscan (Burge and Karlin, 1997), Augustus v2.4 (Stanke and Waack, 2003), GlimmerHMM v3.0.4 (Majoros et al., 2004), GeneID v1.4 (Alioto et al., 2018), and SNAP (Korf, 2004) were used for *de-novo* prediction. The software package GeMoMa v1.3.1 was used for prediction based on homologous species (Keilwagen et al., 2016; Keilwagen et al., 2018). The software packages Hisat v2.0.4 (Kim et al., 2015) and Stringtie v1.2.3 (Pertea et al., 2015) were used for assembly based on reference transcripts, and the software packages TransDecoder v2.0 and GeneMarkS-T v5.1 (Tang et al., 2015) were used for gene prediction. The software package PASA v2.0.2 was used to predict Unigene sequences without reference based on transcriptome data (Campbell et al., 2006). Finally, the software package EVM v1.1.1 (Haas et al., 2008) was used to integrate the prediction results obtained by the above three methods. The predicted gene sequences were compared with NR, KOG, GO, KEGG, TrEMBL, and other functional databases by the software package BLAST v2.2.31 (-evalue 1e-5) (Altschul et al., 1990) to perform KEGG pathway, KOG function, GO function and other genes functional annotation analysis.

### Identification of genomic sequence variation in important genes

The whole-genome assemblies sequences of QG10 were compared with the rice reference genome sequence (Oryza_sativa_MSU7 version) using the software package MUMmer v3 (Kurtz et al., 2004). According to the results from the software package MUMmer, the sequence variations and SVs were further re-called using the software package BLAST. The synteny/inversion comparison were analysis by using GenomeSyn_Win.v1 (Zhou et al., 2022). At the site of each sequence variant, the genotypic information for QG10, Nipponbare, and the elite variety having important genes was called according to the results of the one-to-one alignments. The allelic information of sequence variants was detected based on gff files from the Oryza_sativa_MSU7 version. The software packages ClustalW v1.8.3(Thompson et al., 1994) and BLAST v2.2.31 were used for re-detected the sequence variations and detailed haplotype analyses for the well-characterized genes in rice (Zhao et al., 2018b).

## Results

### Nanopore sequencing and genome assembly

We sequenced QG10 genomic DNA to generate about 73.52Gb of Nanopore sequencing raw data. After data quality control, the clean data volume was 63.23Gb containing 3,279,893 reads with a total of 166.34-fold sequencing depth. The reads with 10 -20 kb and 20 - 30 kb sequencing length were account for 51.56% (**Table 1**). The mean reads length of clean sequencing data was 19.28 kb with an N50 length of 25.64 kb. The clean Nanopore sequencing data was re-corrected with the software package Canu (Koren et al., 2017). Then the third-generation sequencing data was re-corrected with the software package Racon (Vaser et al., 2017) for three rounds. Then, the second-generation data were used for three rounds of correction by Pilon (Walker et al., 2014) software, and the stain was removed according to NT alignment. Finally, we obtained a 380.15 Mb genome sequence with the contig N50 was 12.24 Mb. The completeness estimated by Benchmarking Universal Single-Copy Orthologs (BUSCO) was 98.12%.

**Table 1:** The Reads length distribution of Nanopore sequencing clean data.

Length	ReadsNum	TotalLength	Percent	AveLength
2000-5000	390,870	1,364,569,685	2.15%	3,491.10
5001-10000	506,104	3,711,926,879	5.87%	7,334.31
10001-20000	1,160,503	17,449,235,975	27.59%	15,035.92
20001-30000	620,853	15,157,158,521	23.97%	24,413.44
30001-40000	319,208	10,994,112,170	17.38%	34,441.84
40001-50000	158,764	7,047,880,953	11.14%	44,392.18
50001-60000	73,874	4,015,077,789	6.34%	54,350.35
60001-70000	31,197	2,004,190,092	3.16%	64,243.03
70001-80000	11,756	872,009,657	1.37%	74,175.71
>80000	6,764	616,167,771	0.97%	91,095.17

Length: each length range of reads; ReadsNum: the number of sequences in each length range; TotalLength: indicates the total length of sequences in each length. Percent: indicates the proportion of the number of sequences in each length range to the total number of sequences. AveLength: Average length of sequences in each length range.

### Genome assembly and Hi-C scaffolding

**Figure 2:**
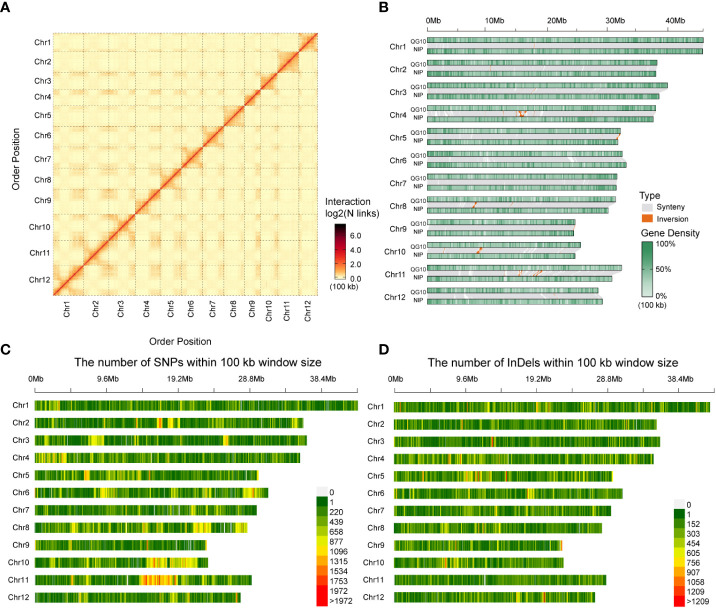
Characteristics of Qigeng10 (QG10) genome and synteny examining, SNPs and InDels mining, Synteny/Inversion comparison with Nipponbare. **(A)**, Visualization of the Hi-C signals in the whole genome of QG10. **(B)**, Chromosomal synteny among QG10 and Nipponbare reference genomes of rice. **(C)**, Distribution of SNPs between QG10 and Nipponbare. **(D)**, Distribution of InDels between QG10 and Nipponbare.

We conducted the assembly in a stepwise fashion following a previously reported approach (Jiang et al., 2020). The Hi-C sequencing raw data was filtered, and the splice sequences and low-quality reads were removed to obtain high-quality clean data. The mapped data was obtained by sequence alignment of clean data with the preliminarily assembled genome. Finally, effective Hi-C data were used for further assembly of the draft genome sequence. LACHESIS software was used for clustering, sorting, and orientating the preliminary assembled genome sequence, and finally, the genomic sequence at the chromosome level was obtained. Finally, we assembled the genome (QG10) into 77 contigs with an N50 length of 11.80 Mb in 27 scaffolds with an N50 length of 30.55 Mb. The assembled genome size was 378.31Mb and the 65 contigs constituted approximately 99.59% of the whole genome. Visualization of the Hi-C signals indicated that 12 square matrix areas in the Hi-C heat map displayed significant differences from the background. These scaffolds were anchored into chromosomes 1–12, respectively (**Figure 2A**; **Table 2**).

**Table 2:** The statistics information of Hi-C assembly data.

Group	Cluster Num	Cluster Len	Order Num	Order Len
Chr1	4	43,382,979	4	43,382,979
Chr2	4	36,173,705	3	36,097,194
Chr3	6	37,757,262	6	37,757,262
Chr4	4	35,918,009	4	35,918,009
Chr5	4	30,375,008	4	30,375,008
Chr6	4	30,649,707	4	30,649,707
Chr7	5	29,809,605	5	29,809,605
Chr8	8	30,022,072	6	29,599,970
Chr9	7	23,219,349	7	23,219,349
Chr10	10	24,170,793	9	24,102,177
Chr11	9	30,667,739	7	30,544,885
Chr12	12	27,708,367	6	26,854,597
Total(Ratio %)	77(96.25)	379854595(99.92)	65(84.42)	378310742(99.59)

According to the whole genome comparison, the genome of QG10 showed four sequence inversions with a length of about 0.5-2 Mb compared with the Nipponbare genome at the position of about 14-16 Mb on chromosome 4, 30-31 Mb on chromosome 5, 5.5-6 Mb on chromosome 8, and 5.5-6 Mb on chromosome 10 (**Figure 2B**). We identified 1,080,819 SNPs and 682,392 InDels between QG10 and Nipponbare on 12 chromosomes (**Figures 2C, D**).

### Genome annotation and repeat analysis

We annotated the repeat regions in our QG10 assembly by Repeat Masker and detected 492,503 repetitive regions with 177.52 Mb repeat length that contained 242,211 Class I retrotransposons, 223,439 Class II DNA transposons, 833 Potential Host Gene, and 2,222 simple sequence repeats (**Table 3**). The repeat regions make up 46.7% of the QG10 assembly genome.

**Table 3:** The statistics information of repeat regions in QG10 assembly.

Type	Number	Length	Rate (%)
Class I	242,211	123,543,619	32.50
Class I/DIRS	5,336	6,334,743	1.67
Class I/LARD	87,163	17,713,596	4.66
Class I/LINE	20,531	5,686,323	1.50
Class I/LTR/Copia	25,386	14,961,322	3.94
Class I/LTR/Gypsy	64,824	68,053,651	17.90
Class I/LTR/Unknown	24,079	13,886,683	3.65
Class I/PLE	1,264	547,500	0.14
Class I/SINE	11,311	2,290,288	0.60
Class I/TRIM	1,493	457,017	0.12
Class I/Unknown	824	603,012	0.16
Class II	223,439	57,701,667	15.18
Class II/Crypton	22	1,816	0.00
Class II/Helitron	21,248	6,000,821	1.58
Class II/MITE	18,642	3,713,793	0.98
Class II/Maverick	256	45,395	0.01
Class II/TIR	123,736	34,429,300	9.06
Class II/Unknown	59,535	16,608,349	4.37
Potential Host Gene	833	431,292	0.11
SSR	2,222	754,226	0.20
Unknown	23,798	5,059,225	1.33
Total	492,503	177,517,691	46.70

Type: repeat sequence type, DIRS: Dictyostelium intermediate repeat sequence; LARD: large retrotransposon derivative; LINE: long interspersed nuclear element; LTR: long terminal repeat; PLE: Penelope-like element; SINE: short interspersed nuclear element; TRIM: terminal-repeat retrotransposons in miniature; MITE: miniature inverted-repeat transposable element; TIR: terminal inverted repeat; SSR: simple sequence repeat. Number: the number of repeats obtained; Length: total length of the predicted repeat sequence; Rate (%): the proportion of repeats in the total genome.

We predicted 39,465 genes by the Ab initio method, 56,999 genes by the Homology-based method, and 24,998 by RNA-seq. Finally, a total of 57,599 genes were integrated with the prediction results obtained by the above three methods by using the software package EVM v1.1.1 (**Table 4**). A total of 723 tRNA, 306 rRNA, 194 miRNA, and 5,392 pseudogenes were also predicted. 94.11% of the genes could be annotated into NR, GO, KOG, KEGG, and other databases (**Table 5**).

**Table 4:** The statistics information of the predicted genes.

Method	Software	Species	Gene number
Ab initio	Genscan	–	36,995
	Augustus	–	33,891
	GlimmerHMM	–	72,642
	GeneID	–	51,130
	SNAP	–	48,464
Homology-based	GeMoMa	Oryza_sativa_9311	47,215
		Oryza_sativa_MSU7	60,679
		Oryza_sativa_rapdb	38,019
		Oryza_sativa_R498	52,424
		Arabidopsis_thaliana	22,123
RNAseq	TransDecoder	–	57,661
	GeneMarkS-T	–	30,053
	PASA	–	34,939
Integration	EVM	–	57,599

Method: The strategy used in gene prediction; Software: Software used for gene prediction; Species: Species. Gene number: The number of genes predicted.

**Table 5:** The statistics information of the annotated genes.

Annotation database	Annotated number	Percentage (%)
COG_Annotation	12,901	22.40
GO_Annotation	38,790	67.34
KEGG_Annotation	9,038	15.69
KOG_Annotation	20,498	35.59
Pfam_Annotation	32,058	55.66
Swissprot_Annotation	24,932	43.29
TrEMBL_Annotation	54,193	94.09
nr_Annotation	54,007	93.76
All_Annotated	54,206	94.11

Annotation database: the annotation database. Annotated number: the number of genes annotated to the corresponding database; Percentage (%): indicates the percentage of genes annotated to the database.

### Sequence variants of the genes controlling grain length

**Figure 3:**
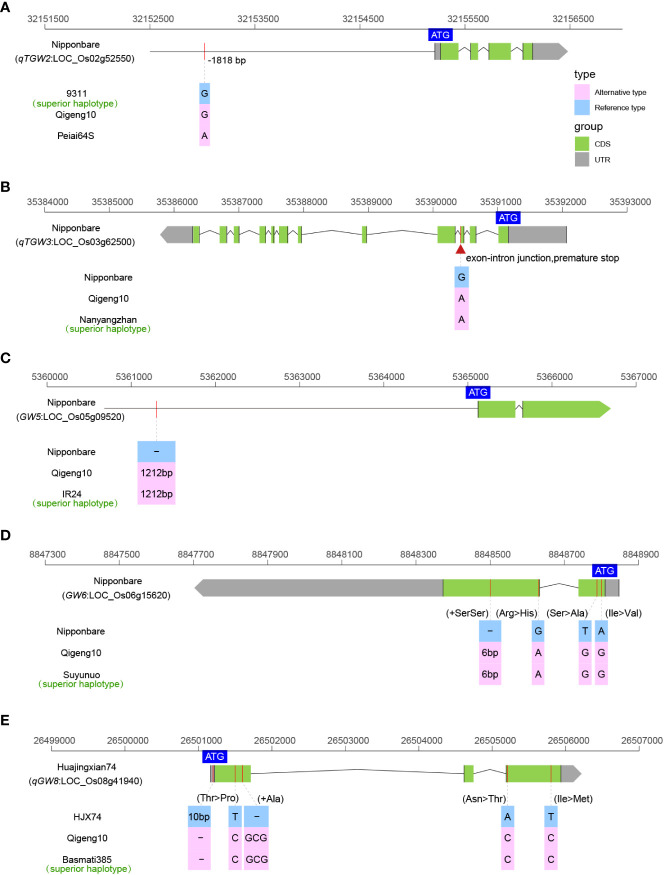
The allelic information of sequence variants in qTGW2 **(A)**, qTGW3 **(B)**, GW5 **(C)**, GW6 **(D)**, and qGW8 **(E)** controlling grain size.

We investigated the sequence variations in 19 cloned genes controlling grain size, including *GW2* (Song et al., 2007), *GS2* (Hu et al., 2015), *qTGW2* (Ruan et al., 2020), *BG1* (Liu et al., 2015), *OsLG3* (Yu et al., 2017), *OsLG3b* (Yu et al., 2018), *GS3* (Fan et al., 2006), *qTGW3* (Hu et al., 2018), *GS5* (Li et al., 2011), *GW5* (Weng et al., 2008), *GS6* (Sun et al., 2013), *GW6* (Shi et al., 2020), *TGW6* (Ishimaru et al., 2013), *GW6a* (Song et al., 2015), *GL6* (Wang et al., 2019), *GLW7* (Si et al., 2016), *GW7* (Wang et al., 2015), *qGW8* (Wang et al., 2012), and *GS9* (Zhao et al., 2018a), to explain the long grain of QG10. A total of five grain size elite alleles (*qTGW2^Nipponbare^*, *qTGW3^Nanyangzhan^*, *GW5^IR24^*, *GW6^Suyunuo^*, and *qGW8^Basmati385^*) were identified controlling grain size in QG10 (**Figure 3**). The *qTGW2* allele in QG10 was identical to Nipponbare having the key variants (G/A) at -1818 bp in the promoter region (**Figure 3A**). The *qTGW3* allele in QG10 had the key splicing-site mutation as Nanyangzhan, which was a long-grain *indica* rice (**Figure 3B**). The *GW5* allele in QG10 was found without the critical loss of the 1,212 bp deletion mutation as IR24 a long narrow grain *indica* rice (**Figure 3C**). The *GW6* allele in QG10 had the key mutation (6 bp) as Suyunuo, which was a wider grain *indica* rice (**Figure 3D**). The *qGW8* allele in QG10 had the five variants as Basmati385, a long narrow grain *indica* rice (**Figure 3E**). Tracing the origin of these genes, it was found that *qTGW3* (Nanyangzhan type), *GW5* (IR24 type), *GW6* (Suyunuo type), and *qGW8* (Basmati385 type) belonged to *indica* subspecies, while *qTGW2* (Nipponbare type) were mainly derived from *japonica* subspecies. In addition, these genes controlling grain type are all rare genotypes in *japonica* rice and have important application value in long grain type *japonica* rice breeding.

### Sequence variants of the genes controlling cold tolerance

**Figure 4:**
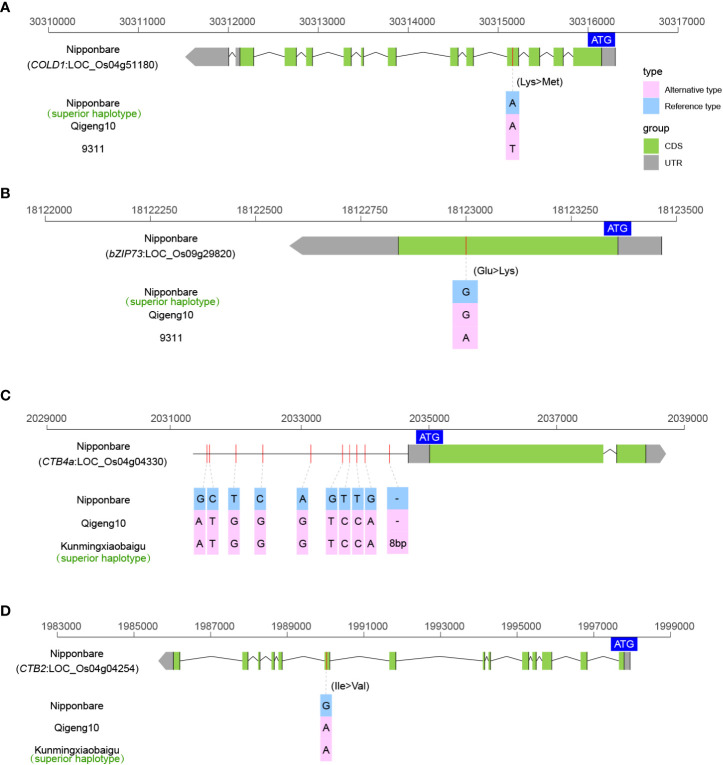
The allelic information of sequence variants in COLD1 **(A)**, bZIP73 **(B)**, CTB4a **(C)**, and CTB2 **(D)** controlling cold tolerance in rice.

Cold tolerance is the key agricultural trait controlling rice production and geographic distribution. We investigated the sequence variations in 10 cloned genes controlling cold tolerance, including *bZIP73* (Liu et al., 2018), *COLD1* (Ma et al., 2015), *Ctb1* (Saito et al., 2010), *CTB2* (Li et al., 2021b), *CTB4a* (Zhang et al., 2017), *HAN1* (Mao et al., 2019), *ltt1* (Xu et al., 2020), *OsLTPL159* (Zhao et al., 2020), *qLTG3-1* (Fujino et al., 2008), and *qPSR10* (Xiao et al., 2018), to explain the high cold tolerance of QG10. A total of four elite alleles (*COLD1^Nipponbare^*, *bZIP73^Nipponbare^*, *CTB4a^Kunmingxiaobaigu^*, and *CTB2^Kunmingxiaobaigu^*) were identified as controlling cold tolerance in QG10 (**Figure 4**). The *COLD1* allele in QG10 was identical to Nipponbare to have the key SNP in the fourth exon region (**Figure 4A**). The *bZIP73* allele in QG10 was found having the key SNP mutation (G/A) as Nipponbare (**Figure 4B**). The *CTB4a* allele in QG10 was found to have the ten mutations as Kunmingxiaobaigu, which was a cold tolerance variety from Yunnan Province (**Figure 4C**). The *CTB2* allele in QG10 was found also have the ten key SNPs as Kunmingxiaobaigu (**Figure 4D**). The *COLD1* (Nipponbare type) and *bZIP73* (Nipponbare type) all belong to *japonica* subspecies. The *CTB4a* (Kunmingxiaobaigu type) and *CTB2* (Kunmingxiaobaigu type) were all rare alleles in Northeast *japonica* rice and have important application value in Northeast *japonica* rice breeding.

### Sequence variants of the genes controlling early heading

**Figure 5:**
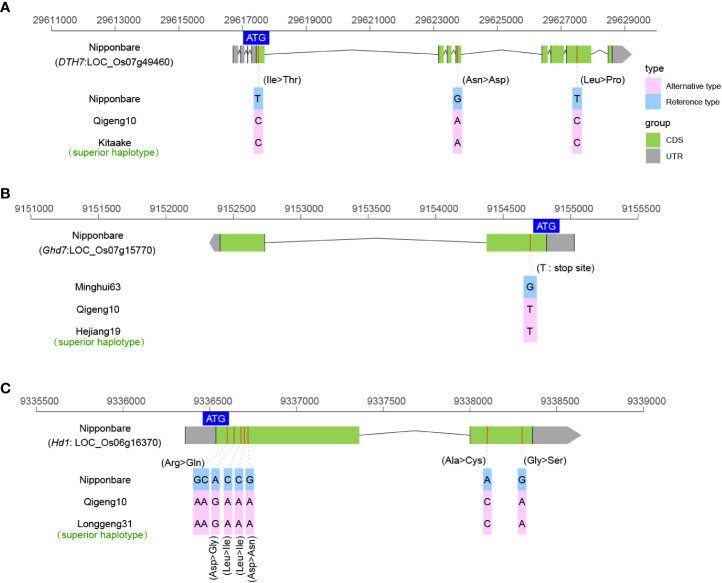
The allelic information of sequence variants in DTH7 **(A)**, Ghd7 **(B)**, and Hd1 **(C)** controlling heading date in rice.

The heading date is one of the most important factors determining rice distribution and the final yield. We investigated the sequence variations in 11 cloned genes related to early heading under long-day conditions, including *DTH7* (Gao et al., 2014), *Ghd7* (Xue et al., 2008), *Ghd8* (Yan et al., 2011), *Ehd1* (Doi et al., 2004), *Ehd3* (Matsubara et al., 2011), *Ehd4* (Gao et al., 2013), *Hd1* (Yano et al., 2000), *Hd3a* (Kojima et al., 2002), *Hd6* (Takahashi et al., 2001), *Hd16* (Hori et al., 2013), and *Hd17* (Matsubara et al., 2012). Among these heading date genes, only the *DTH7*, *Ghd7*, and *Hd1* haplotypes were found to have non-functional alleles. The *DTH7* allele in QG10 was found to have the three mutations as Kitaake, which originated at the northern limit of rice cultivation in Hokkaido, Japan (**Figure 5A**). Kitaake is reported insensitive to day length, short in stature, and completes its life cycle in about 9 weeks (Jain et al., 2019). The *Ghd7* allele in QG10 was found having the critical mutations as Hejiang19, which is an early-maturity rice variety in Heilongjiang Province (**Figure 5B**). The *Hd1* allele in QG10 was found to have the non-functional allele as Longgeng31, which is the major plant rice variety in Heilongjiang Province (**Figure 5C**). These results indicated that the heading gene combinations of *Hd1*, *DTH7*, and *Ghd7* determined the early heading in QG10 in the northernmost province of China.

### Sequence variants of the genes controlling disease resistance

**Figure 6:**
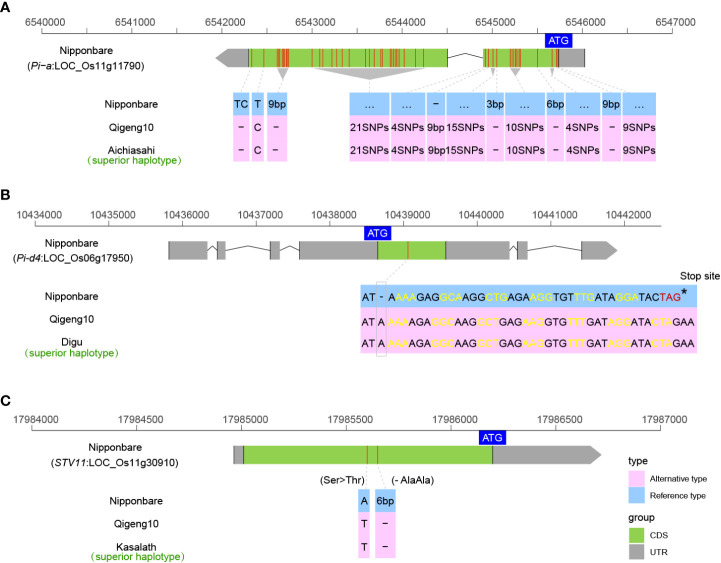
The allelic information of sequence variants in Pia **(A)**, Pid4 **(B)**, and STV11 **(C)** controlling disease resistance in rice.

Blast is one of the most devastating rice diseases in Heilongjiang Province. We investigated the sequence variations in 14 cloned rice blast-resistant genes, including *Pi5* (Lee et al., 2009), *Pi21* (Fukuoka et al., 2009), *Pi36* (Liu et al., 2007), *Pi37* (Lin et al., 2007), *Pi54* (Sharma et al., 2010), *Pi56* (Liu et al., 2013), *Pia* (Okuyama et al., 2011), *Pish* (Takahashi et al., 2010), *Pit* (Hayashi and Yoshida, 2009), *Pita* (Bryan et al., 2000), *Pigm* (Deng et al., 2017), *Pid2* (Chen et al., 2006), *Pid3* (Shang et al., 2009), and *Pid4* (Chen et al., 2018), to explain the blast resistance. Finally, only two blast resistance genes, *Pia* and *Pid4*, were found in QG10. The *Pia* allele in QG10 was found to have the resistant genotype as Akihikari, which encodes a nucleotide-binding site (NBS) and a C-terminal leucine-rich repeat (LRR) domain protein (**Figure 6A**). The *Pid4* allele in QG10 was found to have the resistant genotype as Digu, which encodes a coiled-coil nucleotide-binding site leucine-rich repeat (CC-NBS-LRR) protein (**Figure 6B**). The *Pia* (Akihikari type) was the major blast-resistant gene in Northeast China. The *Pid4* (Digu type) was a rare allele in *japonica* rice and has important application value in Northeast *japonica* rice breeding.

Rice stripe virus (RSV), an RNA virus belonging to the genus *Tenuivirus* and transmitted by small brown planthoppers, causes one of the most destructive rice diseases (Wang et al., 2014). RSV has become more and more serious in Heilongjiang province in recent years. But, almost the majority of *japonica* varieties cultivated in Heilongjiang are highly susceptible to RSV (Wang et al., 2014). The *STV11* was the first cloned resistant gene of RSV, which encodes a sulfotransferase (*OsSOT1*) catalyzing the conversion of salicylic acid (SA) into sulphonated SA (SSA) (Wang et al., 2014). The *STV11* allele in QG10 was found to have the resistant genotype as Kasalath, which is a high-resistance *indica* landrace (**Figure 6C**). The *STV11^QG10^* was a useful resistant gene in *japonica* rice breeding.

### Sequence variants of fragrance, fertilizer use efficiency and other yield related genes

**Figure 7:**
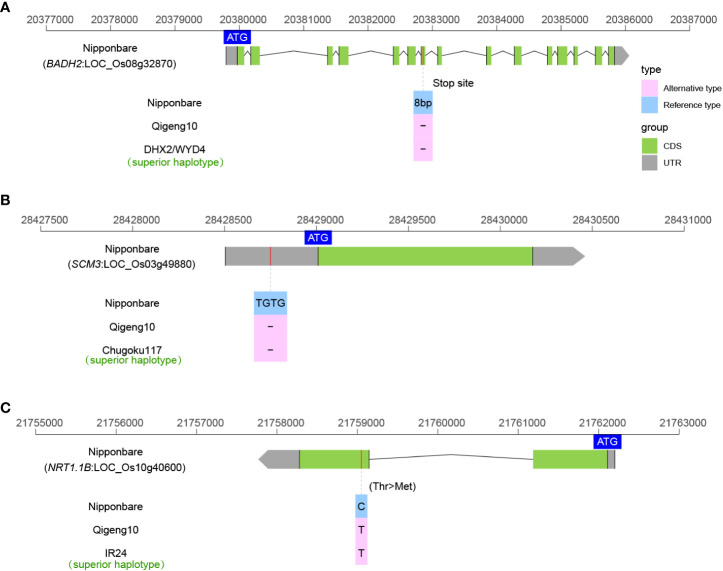
The allelic sequence variants of BADH2 **(A)**, SCM3 **(B)**, and NRT1.1B **(C)** in rice.

Fragrant rice is popular among consumers worldwide because its market price is much higher than that of nonfragrant rice. Fragrant was found to be controlled by *BADH2* in rice (Chen et al., 2008). The *BADH2* in QG10 was the typical *badh2-E2* type of a 7-bp deletion as DHX2 that caused fragrance (**Figure 7A**). We also investigated the sequence variations in three cloned grain number genes (*Gn1a* (Ashikari et al., 2005), *GNP1* (Wu et al., 2016), and *SPIKE* (Fujita et al., 2013)), and three lodging-resistance genes (*Sd1* (Spielmeyer et al., 2002), *SCM2* (Ookawa et al., 2010), and *SCM3* (Yano et al., 2014)). Only the *SCM3* allele in QG10 was found to have the key allele as Chugoku117, which is a stronger culms rice variety in Japan (**Figure 7B**). There was no elite grain number gene found in QG10. *NRT1.1B* is a key gene controlling the nitrogen-use efficiency (NUE) in rice (Hu et al., 2015). The *NRT1.1B* allele in QG10 was found to have the *indica* variation, which has higher nitrate absorption activity (**Figure 7C**).

## Discussion

### Indica genome introgression and large SVs were found in Qigeng10

*Japonica*/*Geng* and *Indica*/*Xian* are the two *major* subspecies of Asian cultivated rice (Zhang et al., 2016a). Owing to long-term differentiation and adaptation, both *Indica* and *Japonica* rice contain many favorable genes. Therefore, combining the favorable genes of the two subspecies has great value for creating genotypes with greater yield potential, stronger stress resistance, and better quality (Gu, 2010). Over the past 50 years, the combination of plant ideotypes and favorable vigor through hybridization between *indica* and *japonica* rice has greatly contributed to yield improvements in modern *japonica* rice in Northeast China (Tang and Chen, 2021). In recent years, a series of high-yielding and good-quality *japonica* cultivars have been obtained from hybridization of *Indica*/*Japonica* and the cultivation area of them was more than 4 million ha in Northeast China (Cui et al., 2022). The new fragrant early *japonica* rice cultivar QG10 was derived from a cross between ‘Wuyoudao4 and Suigeng4’, which were all derived from the hybridization of *Indica*/*Japonica*. In recently, Wang et al. (2023) chose six interrelated modern Chinese temperate *japonica* varieties and six related Japanese *japonica* varieties to investigate genome enhancement in temperate *japonica* varieties during modern breeding. They found many large SVs in Zhonghua11 (ZH11), Liaogeng5 (LG5), and Daohuaxiang2 (DHX2/WYD4). These large-fragment in the same location introgression from *indica* were also found in QG10 on chromosomes 4, 8, and 10 (**Figure 2B**). Several *indica* superior alleles including *qTGW3^Nanyangzhan^*, *GW5^IR24^*, *GW6^Suyunuo^*, *qGW8^Basmati385^*, *Pid4^Digu^*, *STV11^Kasalath^*, *NRT1.1B^IR24^*, and *badh2-E2* were also be found in QG10. This information indicated that the *indica* genome introgression was common in the modern temperate *japonica* rice breeding in Northeast China. Superior alleles were found in Qigeng10 and had important value for breeding in Northeast China

### Superior alleles were found in Qigeng10 and had important value for breeding in Northeast China

**Figure 8:**
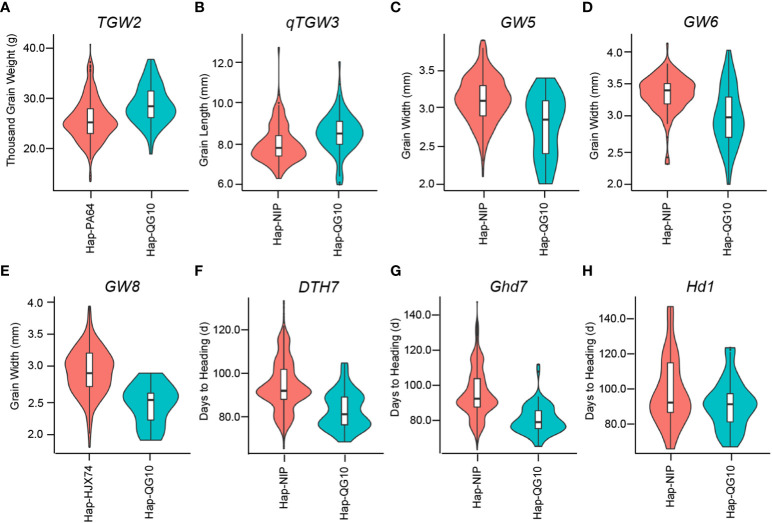
Comparison of grain shape traits and days to heading between different gene haplotypes in 3k panel.

Greater yield potential, stronger stress resistance, and better quality (longer grain and fragrant) are key agronomy traits that directly influence the market price of rice. Consumers in East Asia, including North China, Japan, and Korea tend to prefer longer fragrant *japonica* rice (Lu et al., 2022). So, the longer fragrant *japonica* rice from Northeast China, represented by Daohuaxiang2 (DHX2/WYD4), is the most famous rice in the Chinese market. QG10 was derived from DHX2 and solved some defects of DHX2 including poor lodging resistance, lack of cold tolerance, weak blast resistance, and late maturity. Therefore, the construction of a high-quality genome of ‘QG10’ is essential for further improvement of this cultivar or its progenies, as well as accelerating the process of fragrant *japonica* rice breeding, by providing genomic resources that could be directly applied to fragrant *japonica* rice cultivars. In this study, we found five superior alleles (*qTGW2^Nipponbare^*, *qTGW3^Nanyangzhan^*, *GW5^IR24^*, *GW6^Suyunuo^*, and *qGW8^Basmati385^*) controlling long grain size in QG10. To compare the phenotype of different gene haplotypes in rice germplasm, we investigated the grain shape traits and days to heading of 3k cultivars in the website (https://www.rmbreeding.cn/)(Wang et al., 2020). The results showed that the functional haplotype of QG10 controlling longer and slider grain (**Figures 8A-E**) and early heading (Figure 8f-h). Most of them was belong to the rare alleles for controlling longer grain and were less application in rice breeding in Northeast China. The blast resistant alleles (*Pid4^Digu^*), RSV allele (*STV11^Kasalath^*), and NUE allele (*NRT1.1B^IR24^*) were also belong to the rare alleles for rice breeding in Northeast China. In the future, we will develop molecular assisted markers for the improvement of *japonica* rice varieties in Northeast China, and expanded the gene pool of *japonica* rice in Northeast China.

## Conclusions

In this study, we present chromosome-level genome assembly of an early-matured aromatic long-grain *japonica* rice variety Qigeng10 by using a combination of Nanopore and Hi-C platforms. The total assembly size is 378.31Mb with an N50 length of 30.55 Mb. A total of 18 superior haplotypes including five long-grain alleles (*qTGW2^Nipponbare^*, *qTGW3^Nanyangzhan^*, *GW5^IR24^*, *GW6^Suyunuo^*, and *qGW8^Basmati385^*), four cold tolerant alleles (*COLD1^Nipponbare^*, *bZIP73^Nipponbare^*, *CTB4a^Kunmingxiaobaigu^*, and *CTB2^Kunmingxiaobaigu^*), three non-functional heading date alleles (*DTH7^Kitaake^*, *Ghd7^Hejiang19^*, and *Hd1^Longgeng31^*), two blast resistant alleles (*Pia ^Akihikari^* and *Pid4^Digu^*), a rice stripe virus resistant allele *STV11^Kasalath^*, a higher nitrate absorption allele *NRT1.1B^IR24^*, a lodging resistant allele *SCM3^Chugoku117^*, and the typical aromatic allele *badh2-E2*, were identified in QG10. This information will accelerate the process of fragrant *japonica* rice breeding in Northeast China, by providing genomic resources that could be directly applied to fragrant *japonica* rice cultivars or development of molecular assisted markers for the improvement of *japonica* rice varieties.

## Data availability statement

The data presented in the study are deposited in the Data Center of Beijing Institute of Genomics(Big) repository, accession number WGS029943 (PRJCA013131; SAMC988458).

## Author contributions

Conceptualization: SJ and JW. Methodology: SJ. Software: XZ, XY, BM. Validation: SJ, HJ and YW. Formal analysis: JH and KT. Investigation: CL and LW. Resources: JW. Data curation: SJ. Writing—original draft preparation: SJ. Writing—review and editing: SJ. Visualization: SJ. Project administration: SJ. Funding acquisition: SJ and XZ. All authors contributed to the article and approved the submitted version.

## References

[B1] Alioto T. Blanco E. Parra G. Guigó R. (2018). Using geneid to identify genes. Curr. Protoc. Bioinf. 64, e56. doi: 10.1002/cpbi.56 30332532

[B2] Altschul S. Gish W. Miller W. Myers E. Lipman D. (1990). Basic local alignment search tool. J. Mol. Biol. 215, 403–410. doi: 10.1016/S0022-2836(05)80360-2 2231712

[B3] Ashikari M. Sakakibara H. Lin S. Yamamoto T. Takashi T. Nishimura A.. (2005). Cytokinin oxidase regulates rice grain production. Science 309, 741–745. doi: 10.1126/science.1113373 15976269

[B4] Bryan G. Wu K. Farrall L. Jia Y. Hershey H. Mcadams S.. (2000). A single amino acid difference distinguishes resistant and susceptible alleles of the rice blast resistance gene pi-ta. Plant Cell 12, 2033–2046. doi: 10.2307/3871103 11090207 PMC150156

[B5] Burge C. Karlin S. (1997). Prediction of complete gene structures in human genomic DNA. J. Mol. Biol. 268, 78–94. doi: 10.1006/jmbi.1997.0951 9149143

[B6] Burton J. Adey A. Patwardhan R. Qiu R. Kitzman J. Shendure J. (2013). Chromosome-scale scaffolding of *de novo* genome assemblies based on chromatin interactions. Nat. Biotechnol. 31, 1119–1125. doi: 10.1038/nbt.2727 24185095 PMC4117202

[B7] Campbell M. Haas B. Hamilton J. Mount S. Buell C. (2006). Comprehensive analysis of alternative splicing in rice and comparative analyses with arabidopsis. BMC Genomics 7, 327. doi: 10.1186/1471-2164-7-327 17194304 PMC1769492

[B8] Chen X. Shang J. Chen D. Lei C. Zou Y. Zhai W.. (2006). A b-lectin receptor kinase gene conferring rice blast resistance. Plant J. 46, 794–804. doi: 10.1111/j.1365-313X.2006.02739.x 16709195

[B9] Chen S. Yang Y. Shi W. Ji Q. He F. Zhang Z.. (2008). Badh2, encoding betaine aldehyde dehydrogenase, inhibits the biosynthesis of 2-acetyl-1-pyrroline, a major component in rice fragrance. Plant Cell 20, 1850–1861. doi: 10.1105/tpc.108.058917 18599581 PMC2518245

[B10] Chen Z. Zhao W. Zhu X. Zou C. Yin J. Chern M.. (2018). Identification and characterization of rice blast resistance gene Pid4 by a combination of transcriptomic profiling and genome analysis. J. Genet. Genomics 45, 663–672. doi: 10.1016/j.jgg.2018.10.007 30606471

[B11] Choi J. Lye Z. Groen S. Dai X. Rughani P. Zaaijer S.. (2020). Nanopore sequencing-based genome assembly and evolutionary genomics of circum-basmati rice. Genome Biol. 21, 21. doi: 10.1186/s13059-020-1938-2 32019604 PMC7001208

[B12] Cui D. Zhou H. Ma X. Lin Z. Sun L. Han B.. (2022). Genomic insights on the contribution of introgressions from Xian/Indica to the genetic improvement of Geng/Japonica rice cultivars. Plant Commun. 3, 100325. doi: 10.1016/j.xplc.2022.100325 35576158 PMC9251437

[B13] Deng Y. Zhai K. Xie Z. Yang D. Zhu X. Liu J.. (2017). Epigenetic regulation of antagonistic receptors confers rice blast resistance with yield balance. Science 355, 962–965. doi: 10.1126/science.aai8898 28154240

[B14] Doi K. Izawa T. Fuse T. Yamanouchi U. Kubo T. Shimatani Z.. (2004). Ehd1, a b-type response regulator in rice, confers short-day promotion of flowering and controls FT-like gene expression independently of Hd1. Genes Dev. 18, 926–936. doi: 10.1101/gad.1189604 15078816 PMC395851

[B15] Du H. Yu Y. Ma Y. Gao Q. Cao Y. Chen Z.. (2017). Sequencing and *de novo* assembly of a near complete indica rice genome. Nat. Commun. 8, 15324. doi: 10.1038/ncomms15324 28469237 PMC5418594

[B16] Fan C. Xing Y. Mao H. Lu T. Han B. Xu C.. (2006). GS3, a major QTL for grain length and weight and minor QTL for grain width and thickness in rice, encodes a putative transmembrane protein. Theor. Appl. Genet. 112, 1164–1171. doi: 10.1007/s00122-006-0218-1 16453132

[B17] Fujino K. Sekiguchi H. Matsuda Y. Sugimoto K. Ono K. Yano M. (2008). Molecular identification of a major quantitative trait locus, qLTG3-1, controlling low-temperature germinability in rice. Proc. Natl. Acad. Sci. U.S.A. 105, 12623–12628. doi: 10.1073/pnas.0805303105 18719107 PMC2527961

[B18] Fujita D. Trijatmiko K. Tagle A. Sapasap M. Koide Y. Sasaki K.. (2013). NAL1 allele from a rice landrace greatly increases yield in modern indica cultivars. Proc. Natl. Acad. Sci. U.S.A. 110, 20431–20436. doi: 10.1073/pnas.1310790110 24297875 PMC3870739

[B19] Fukuoka S. Saka N. Koga H. Ono K. Shimizu T. Ebana K.. (2009). Loss of function of a proline-containing protein confers durable disease resistance in rice. Aug 21. Science 325, 998–1001. doi: 10.1126/science.1175550 19696351

[B20] Gao H. Jin M. Zheng X. Chen J. Yuan D. Xin Y.. (2014). Days to heading 7, a major quantitative locus determining photoperiod sensitivity and regional adaptation in rice. Proc. Natl. Acad. Sci. U.S.A. 111, 16337–16342. doi: 10.1073/pnas.1418204111 25378698 PMC4246261

[B21] Gao H. Zhao B. Zhang J. Song L. Wu H. Yu Y.. (2012). High-quality and high-yield cultivation techniques of a new high-quality rice variety wuyoudao no. 4. Heilongjiang Agric. Sci. 154-155.

[B22] Gao H. Zheng X. Fei G. Chen J. Jin M. Ren Y.. (2013). Ehd4 encodes a novel and oryza-genus-specific regulator of photoperiodic flowering in rice. PloS Genet. 9, e1003281. doi: 10.1371/journal.pgen.1003281 23437005 PMC3578780

[B23] Goff S. Ricke D. Lan T. Presting G. Wang R. Dunn M.. (2002). A draft sequence of the rice genome (Oryza sativa l. ssp. japonica). Science 296, 92–100. doi: 10.1126/science.1068275 11935018

[B24] Gross B. Zhao Z. (2014). Archaeological and genetic insights into the origins of domesticated rice. Proc. Natl. Acad. Sci. U.S.A. 111, 6190–6197. doi: 10.1073/pnas.1308942110 24753573 PMC4035933

[B25] Gu H. (2010). Discussion on the aspects of high-yielding breeding in rice. Acta Agronomica Sin. 36, 1431–1439.

[B26] Gu H. Liang S. Zhao J. (2022). Novel sequencing and genomic technologies revolutionized rice genomic study and breeding. Agronomy 12, 218. doi: 10.3390/agronomy12010218

[B27] Haas B. Salzberg S. Zhu W. Pertea M. Allen J. Orvis J.. (2008). Automated eukaryotic gene structure annotation using EVidenceModeler and the program to assemble spliced alignments. Genome Biol. 9, R7. doi: 10.1186/gb-2008-9-1-r7 18190707 PMC2395244

[B28] Hayashi K. Yoshida H. (2009). Refunctionalization of the ancient rice blast disease resistance gene pit by the recruitment of a retrotransposon as a promoter. Plant J. 57, 413–425. doi: 10.1111/j.1365-313X.2008.03694.x 18808453

[B29] Hoede C. Arnoux S. Moisset M. Chaumier T. Inizan O. Jamilloux V.. (2014). PASTEC: an automatic transposable element classification tool. PloS One 9, e91929. doi: 10.1371/journal.pone.0091929 24786468 PMC4008368

[B30] Hori K. Ogiso-Tanaka E. Matsubara K. Yamanouchi U. Ebana K. Yano M. (2013). Hd16, a gene for casein kinase I, is involved in the control of rice flowering time by modulating the day-length response. Plant J. 76, 36–46. doi: 10.1111/tpj.12268 23789941 PMC4223384

[B31] Hu Z. Lu S. Wang M. He H. Sun L. Wang H.. (2018). A novel QTL qTGW3 encodes the GSK3/SHAGGY-like kinase OsGSK5/OsSK41 that interacts with OsARF4 to negatively regulate grain size and weight in rice. Mol. Plant 11, 736–749. doi: 10.1016/j.molp.2018.03.005 29567449

[B32] Hu J. Wang Y. Fang Y. Zeng L. Xu J. Yu H.. (2015). A rare allele of GS2 enhances grain size and grain yield in rice. Mol. Plant 8, 1455–1465. doi: 10.1016/j.molp.2015.07.002 26187814

[B33] Hu B. Wang W. Ou S. Tang J. Li H. Che R.. (2015). Variation in NRT1.1B contributes to nitrate-use divergence between rice subspecies. Nat. Genet. 47, 834–838. doi: 10.1038/ng.3337 26053497

[B34] Hui S. Li H. Mawia A. Zhou L. Cai J. Ahmad S.. (2022). Production of aromatic three-line hybrid rice using novel alleles of BADH2. Plant Biotechnol. J. 20, 59–74. doi: 10.1111/pbi.13695 34465003 PMC8710899

[B35] International-Rice-Genome-Sequencing-Project (2005). The map-based sequence of the rice genome. Nature 436, 793–800. doi: 10.1038/nature03895 16100779

[B36] Ishimaru K. Hirotsu N. Madoka Y. Murakami N. Hara N. Onodera H.. (2013). Loss of function of the IAA-glucose hydrolase gene TGW6 enhances rice grain weight and increases yield. Nat. Genet. 45, 707–711. doi: 10.1038/ng.2612 23583977

[B37] Jain R. Jenkins J. Shu S. Chern M. Martin J. Copetti D.. (2019). Genome sequence of the model rice variety KitaakeX. BMC Genomics 20, 905. doi: 10.1186/s12864-019-6262-4 31775618 PMC6882167

[B38] Jiang S. An H. Xu F. Zhang X. (2020). Chromosome-level genome assembly and annotation of the loquat (Eriobotrya japonica) genome. Gigascience 9, giaa015. doi: 10.1093/gigascience/giaa015 32141509 PMC7059265

[B39] Jurka J. Kapitonov V. Pavlicek A. Klonowski P. Kohany O. Walichiewicz J. (2005). Repbase update, a database of eukaryotic repetitive elements. Cytogenetic Genome Res. 110, 462–467. doi: 10.1159/000084979 16093699

[B40] Keilwagen J. Hartung F. Paulini M. Twardziok S. Grau J. (2018). Combining RNA-seq data and homology-based gene prediction for plants, animals and fungi. BMC Bioinf. 19, 189. doi: 10.1186/s12859-018-2203-5 PMC5975413 29843602

[B41] Keilwagen J. Wenk M. Erickson J. Schattat M. Jan G. Frank H. (2016). Using intron position Conserv. homology-based Gene prediction. Nucleic Acids Res. 44, e89. doi: 10.1093/nar/gkw092 26893356 PMC4872089

[B42] Kim D. Langmead B. Salzberg S. (2015). HISAT: a fast spliced aligner with low memory requirements. Nat. Methods 12, 357–360. doi: 10.1038/nmeth.3317 25751142 PMC4655817

[B43] Kojima S. Takahashi Y. Kobayashi Y. Monna L. Sasaki T. Araki T.. (2002). Hd3a, a rice ortholog of the arabidopsis FT gene, promotes transition to flowering downstream of Hd1 under short-day conditions. Plant Cell Physiol. 43, 1096–1105. doi: 10.1093/pcp/pcf156 12407188

[B44] Koren S. Walenz B. Berlin K. Miller J. Bergman N. Phillippy A. (2017). Canu: scalable and accurate long-read assembly *via* adaptive k-mer weighting and repeat separation. Genome Res. 27, 722–736. doi: 10.1101/gr.215087.116 28298431 PMC5411767

[B45] Korf I. (2004). Gene finding in novel genomes. BMC Bioinf. 5. doi: 10.1186/1471-2105-5-59 PMC421630 15144565

[B46] Kurtz S. Phillippy A. Delcher A. Smoot M. Shumway M. Antonescu C.. (2004). Versatile and open software for comparing large genomes. Genome Biol. 5, R12. doi: 10.1186/gb-2004-5-2-r12 14759262 PMC395750

[B47] Lee S. K. Song M. Y. Seo Y. S. Kim H. K. KO. S. Cao P. J.. (2009). Rice Pi5-mediated resistance to magnaporthe oryzae requires the presence of two coiled-coil-nucleotide-binding-leucine-rich repeat genes. Genetics 181, 1627–1638. doi: 10.1534/genetics.108.099226 19153255 PMC2666525

[B48] Li H. Durbin R. (2009). Fast and accurate short read alignment with burrows-wheeler transform. Bioinformatics 25, 1754–1760. doi: 10.1093/bioinformatics/btp324 19451168 PMC2705234

[B49] Li Y. Fan C. Xing Y. Jiang Y. Luo L. Sun L.. (2011). Natural variation in GS5 plays an important role in regulating grain size and yield in rice. Nat. Genet. 43, 1266–1269. doi: 10.1038/ng.977 22019783

[B50] Li F. Gao Y. Wu B. Cai Q. Zhan P. Yang W.. (2021a). High-quality *de novo* genome assembly of huajingxian 74, a receptor parent of single segment substitution lines. Rice Sci. 28, 109–113. doi: 10.1016/j.rsci.2020.09.010

[B51] Li G. Jain R. Chern M. Pham N. Martin J. Wei T.. (2017). The sequences of 1504 mutants in the model rice variety kitaake facilitate rapid functional genomic studies. Plant Cell Physiol. 29, 1218–1231. doi: 10.1105/tpc.17.00154 PMC5502455 28576844

[B52] Li X. Wu L. Wang J. Sun J. Xia X. Geng X.. (2018). Genome sequencing of rice subspecies and genetic analysis of recombinant lines reveals regional yield- and quality-associated loci. BMC Biol. 16, 102. doi: 10.1186/s12915-018-0572-x 30227868 PMC6145349

[B53] Li J. Zeng Y. Pan Y. Zhou L. Zhang Z. Guo H.. (2021b). Stepwise selection of natural variations at CTB2 and CTB4a improves cold adaptation during domestication of japonica rice. New Phytol. 231, 1056–1072. doi: 10.1111/nph.17407 33892513

[B54] Lin F. Chen S. Que Z. Wang L. Liu X. Pan Q. (2007). The blast resistance gene Pi37 encodes a nucleotide binding site leucine-rich repeat protein and is a member of a resistance gene cluster on rice chromosome 1. Genetics 177, 1871–1880. doi: 10.1534/genetics.107.080648 17947408 PMC2147969

[B55] Lin B. Hui J. Mao H. (2021). Nanopore technology and its applications in gene sequencing. Biosensors 11, 214. doi: 10.3390/bios11070214 34208844 PMC8301755

[B56] Liu X. Lin F. Wang L. Pan Q. (2007). The in silico map-based cloning of Pi36, a rice coiled-coil nucleotide-binding site leucine-rich repeat gene that confers race-specific resistance to the blast fungus. Genetics 176, 2541–2549. doi: 10.1534/genetics.107.075465 17507669 PMC1950653

[B57] Liu Y. Liu B. Zhu X. Yang J. Bordeos A. Wang G.. (2013). Fine-mapping and molecular marker development for Pi56(t), a NBS-LRR gene conferring broad-spectrum resistance to magnaporthe oryzae in rice. Theor. Appl. Genet. 126, 985–998. doi: 10.1007/s00122-012-2031-3 23400829

[B58] Liu C. Ou S. Mao B. Tang J. Wang W. Wang H.. (2018). Early selection of bZIP73 facilitated adaptation of japonica rice to cold climates. Nat. Commun. 9, 3302. doi: 10.1038/s41467-018-05753-w 30120236 PMC6098049

[B59] Liu L. Tong H. Xiao Y. Che R. Xu F. Hu B.. (2015). Activation of big Grain1 significantly improves grain size by regulating auxin transport in rice. Proc. Natl. Acad. Sci. U.S.A. 112, 11102–11107. doi: 10.1073/pnas.1512748112 26283354 PMC4568269

[B60] Lu R L. J. Wang X. Song Z. Ji X. Li N. Ma G.. (2022). Chromosome-level genome assembly of a fragrant japonica rice cultivar ‘Changxianggeng 1813’ provides insights into genomic variations between fragrant and non-fragrant japonica rice. Int. J. Mol. Sci. 23, 9705. doi: 10.3390/ijms23179705 36077110 PMC9456513

[B61] Ma Y. Dai X. Xu Y. Luo W. Zheng X. Zeng D.. (2015). COLD1 confers chilling tolerance in rice. Cell 160, 1209–1221. doi: 10.1016/j.cell.2015.01.046 25728666

[B62] Mahesh H. Shirke M. Singh S. Rajamani A. Hittalmani S. Wang G.. (2016). Indica rice genome assembly, annotation and mining of blast disease resistance genes. BMC Genomics 242. doi: 10.1186/s12864-016-2523-7 PMC4793524 26984283

[B63] Majoros W. Pertea M. Salzberg S. (2004). TigrScan and GlimmerHMM: Two open source ab initio eukaryotic gene-finders. Bioinformatics 20, 2878–2879. doi: 10.1093/bioinformatics/bth315 15145805

[B64] Mao D. Xin Y. Tan Y. Hu X. Bai J. Liu Z.. (2019). Natural variation in the HAN1 gene confers chilling tolerance in rice and allowed adaptation to a temperate climate. Proc. Natl. Acad. Sci. U.S.A. 116, 3494–3501. doi: 10.1073/pnas.1819769116 30808744 PMC6397538

[B65] Matsubara K. Ogiso-Tanaka E. Hori K. Ebana K. Ando T. Yano M. (2012). Natural variation in Hd17, a homolog of arabidopsis ELF3 that is involved in rice photoperiodic flowering. Plant Cell Physiol. 53, 709–716. doi: 10.1093/pcp/pcs028 22399582

[B66] Matsubara K. Yamanouchi U. Nonoue Y. Sugimoto K. Wang Z. Minobe Y.. (2011). Ehd3, encoding a plant homeodomain finger-containing protein, is a critical promoter of rice flowering. Plant J. 66, 603–612. doi: 10.1111/j.1365-313X.2011.04517.x 21284756

[B67] Nie S. Liu Y. Wang C. Gao S. Xu T. Liu Q.. (2017). Assembly of an early-matured japonica (Geng) rice genome, Suijing18, ased on PacBio and illumina sequencing. Sci. Data 170195. doi: 10.1038/sdata.2017.195 PMC5735919 29257136

[B68] Okuyama Y. Kanzaki H. Abe A. Yoshida K. Tamiru M. Saitoh H.. (2011). A multifaceted genomics approach allows the isolation of the rice pia-blast resistance gene consisting of two adjacent NBS-LRR protein genes. Plant J. 66, 467–479. doi: 10.1111/j.1365-313X.2011.04502.x 21251109

[B69] Ookawa T. Hobo T. Yano M. Murata K. Ando T. Miura H.. (2010). New approach for rice improvement using a pleiotropic QTL gene for lodging resistance and yield. Nat. Commun. 1, 132. doi: 10.1038/ncomms1132 21119645 PMC3065348

[B70] Panibe J. Wang L. Li J. Li M. Lee Y. Wang C.. (2021). Chromosomal-level genome assembly of the semi-dwarf rice taichung native 1, an initiator of green revolution. Genomics 113, 2656–2674. doi: 10.1016/j.ygeno.2021.06.006 34111524

[B71] Pertea M. Pertea G. M. Antonescu C. M. Chang T. C. Mendell J. T. Salzberg Sl (2015). StringTie enables improved reconstruction of a transcriptome from RNA-seq reads. Nat. Biotechnol. 33, 290–295. doi: 10.1038/nbt.3122 25690850 PMC4643835

[B72] Price A. Jones N. Pa. P. (2005). *De novo* identification of repeat families in large genomes. Bioinformatics 21, i351–i358. doi: 10.1093/bioinformatics/bti1018 15961478

[B73] Qin P. Lu H. Du H. Wang H. Chen W. Chen Z.. (2021). Pan-genome analysis of 33 genetically diverse rice accessions reveals hidden genomic variations. Cell 184, 3542–3558. doi: 10.1016/j.cell.2021.04.046 34051138

[B74] Ruan B. Shang L. Zhang B. Hu J. Wang Y. Lin H.. (2020). Natural variation in the promoter of TGW2 determines grain width and weight in rice. New Phytol. 227, 629–640. doi: 10.1111/nph.16540 32167575

[B75] Saito K. Hayano-Saito Y. Kuroki M. Sato Y. (2010). Map-based cloning of the rice cold tolerance gene Ctb1. Plant Sci. 179, 97–102. doi: 10.1016/j.plantsci.2010.04.004

[B76] Servant N. Varoquaux N. Lajoie B. Viara E. Chen C. Vert J.. (2015). HiC-pro: An optimized and flexible pipeline for Hi-c data processing. Genome Biol. 16, 259. doi: 10.1186/s13059-015-0831-x 26619908 PMC4665391

[B77] Shang L. Li X. He H. Yuan Q. Song Y. Wei Z.. (2022). A super pan-genomic landscape of rice. Cell Res. 32, 878–896. doi: 10.1038/s41422-022-00685-z 35821092 PMC9525306

[B78] Shang J. Tao Y. Chen X. Zou Y. Lei C. Wang J.. (2009). Identification of a new rice blast resistance gene, Pid3, by genomewide comparison of paired nucleotide-binding site–leucine-rich repeat genes and their pseudogene alleles between the two sequenced rice genomes. Genetics 182, 1303–1311. doi: 10.1534/genetics.109.102871 19506306 PMC2728867

[B79] Sharma T. Rai A. Gupta S. Singh N. (2010). Broad-spectrum blast resistance gene pi-kh cloned from rice line tetep designated as Pi54. J. Plant Biochem. Biotechnol. 19, 87–89. doi: 10.1007/BF03323441

[B80] Shi C. Dong N. Guo T. Ye W. Shan J. Lin H. (2020). A quantitative trait locus GW6 controls rice grain size and yield through the gibberellin pathway. Plant J. 103, 1174–1188. doi: 10.1111/tpj.14793 32365409

[B81] Si L. Chen J. Huang X. Gong H. Luo J. Hou Q.. (2016). OsSPL13 controls grain size in cultivated rice. Nat. Genet. 48, 447–456. doi: 10.1038/ng.3518 26950093

[B82] Simão F. Waterhouse R. Ioannidis P. Kriventseva E. Zdobnov E. (2015). BUSCO: assessing genome assembly and annotation completeness with single-copy orthologs. Bioinformatics 31, 3210–3212. doi: 10.1093/bioinformatics/btv351 26059717

[B83] Song X. Huang W. Shi M. Zhu M. Lin H. (2007). A QTL for rice grain width and weight encodes a previously unknown RING-type E3 ubiquitin ligase. Nat. Genet. 39, 623–630. doi: 10.1038/ng2014 17417637

[B84] Song X. Kuroha T. Ayano M. Furuta T. Nagai K. Komeda N.. (2015). Rare allele of a previously unidentified histone H4 acetyltransferase enhances grain weight, yield, and plant biomass in rice. Proc. Natl. Acad. Sci. U.S.A. 112, 76–81. doi: 10.1073/pnas.1421127112 25535376 PMC4291654

[B85] Spielmeyer W. Ellis M. Chandler P. (2002). Semidwarf (sd-1), “green revolution” rice, contains a defective gibberellin 20-oxidase gene. Proc. Natl. Acad. Sci. U.S.A. 99, 9043–9048. doi: 10.1073/pnas.132266399 12077303 PMC124420

[B86] Stanke M. Waack S. (2003). Gene prediction with a hidden Markov model and a new intron submodel. Bioinformatics 19, ii215–ii225. doi: 10.1093/bioinformatics/btg1080 14534192

[B87] Stein J. Yu Y. Copetti D. Zwickl D. Zhang L. Zhang C.. (2018). Genomes of 13 domesticated and wild rice relatives highlight genetic conservation, turnover and innovation across the genus oryza. Nat. Genet. 50, 285–296. doi: 10.1038/s41588-018-0040-0 29358651

[B88] Sun L. Li X. Fu Y. Zhu Z. Tan L. Liu F.. (2013). GS6, a member of the GRAS gene family, negatively regulates grain size in rice. J. Integr. Plant Biol. 55, 938–949. doi: 10.1111/jipb.12062 23650998

[B89] Takahashi A. Hayashi N. Miyao A. Hirochika H. (2010). Unique features of the rice blast resistance pish locus revealed by large scale retrotransposon-tagging. BMC Plant Biol. 10, 175. doi: 10.1186/1471-2229-10-175 20707904 PMC3017791

[B90] Takahashi Y. Shomura A. Sasaki T. Yano M. (2001). Hd6, a rice quantitative trait locus involved in photoperiod sensitivity, encodes the alpha subunit of protein kinase CK2. Proc. Natl. Acad. Sci. U.S.A. 98, 7922–7927. doi: 10.1073/pnas.111136798 11416158 PMC35444

[B91] Tanaka T. Nishijima R. Teramoto S. Kitomi Y. Hayashi T. Uga Y.. (2020). *De novo* genome assembly of the indica rice variety IR64 using linked-read sequencing and nanopore sequencing. G3 (Bethesda) 10, 1495–1501. doi: 10.1534/g3.119.400871 32184372 PMC7202035

[B92] Tang L. Chen W. (2021). Development trend and prospect of geng rice in northeast China. China Rice, 28(5), 1–4.

[B93] Tang S. Lomsadze A. Borodovsky M. (2015). Identification of protein coding regions in RNA transcripts. Nucleic Acids Res. 43, e78. doi: 10.1093/nar/gkv227 25870408 PMC4499116

[B94] Tarailo-Graovac M. Chen N. (2009). Using RepeatMasker to identify repetitive elements in genomic sequences. Curr. Protoc. Bioinf. 4 (10), 11–14. doi: 10.1002/0471250953.bi0410s25 19274634

[B95] Thompson J. Higgins D. Gibson T. (1994). CLUSTAL W: improving the sensitivity of progressive multiple sequence alignment through sequence weighting, position-specific gap penalties and weight matrix choice. Nucleic Acids Res. 22, 4673–4680. doi: 10.1093/nar/22.22.4673 7984417 PMC308517

[B96] Van Berkum N. Lieberman-Aiden E. Williams L. Imakaev M. Gnirke A. Mirny L. A.. (2010). Hi-C: A method to study the three-dimensional architecture of genomes. J. Vis. Exp. 39, 1869. doi: 10.3791/1869 PMC3149993 20461051

[B97] Vaser R. Sović I. Nagarajan N. Šikić M. (2017). Fast and accurate *de novo* genome assembly from long uncorrected reads. Genome Res. 27, 737–746. doi: 10.1101/gr.214270.116 28100585 PMC5411768

[B98] Walker B. Abeel T. Shea T. Priest M. Abouelliel A. Sakthikumar S.. (2014). Pilon: An integrated tool for comprehensive microbial variant detection and genome assembly improvement. PloS One 9, e112963. doi: 10.1371/journal.pone.0112963 25409509 PMC4237348

[B99] Wang A. Hou Q. Si L. Huang X. Luo J. Lu D.. (2019). The PLATZ transcription factor GL6 affects grain length and number in rice. Plant Physiol. 180, 2077–2090. doi: 10.1104/pp.18.01574 31138620 PMC6670106

[B100] Wang S. Li S. Liu Q. Wu K. Zhang J. Wang S.. (2015). The OsSPL16-GW7 regulatory module determines grain shape and simultaneously improves rice yield and grain quality. Nat. Genet. 47, 949–954. doi: 10.1038/ng.3352 26147620

[B101] Wang Y. Li F. Zhang F. Wu L. Xu N. Sun Q.. (2023). Time-ordering japonica/geng genomes analysis indicates the importance of large structural variants in rice breeding. Plant Biotechnol. J. 21, 202–218. doi: 10.1111/pbi.13938 36196761 PMC9829401

[B102] Wang Q. Liu Y. He J. Zheng X. Hu J. Liu Y.. (2014). STV11 encodes a sulphotransferase and confers durable resistance to rice stripe virus. Nat. Commun. 5. doi: 10.1038/ncomms5768 PMC4164775 25203424

[B103] Wang S. Wu K. Yuan Q. Liu X. Liu Z. Lin X.. (2012). Control of grain size, shape and quality by OsSPL16 in rice. Nat. Genet. 44, 950–954. doi: 10.1038/ng.2327 22729225

[B104] Wang C. Yu H. Huang J. Wang W. Faruquee M. Zhang F.. (2020). Towards a deeper haplotype mining of complex traits in rice with RFGB v2.0. Plant Biotechnol. J. 18, 14–16. doi: 10.1111/pbi.13215 31336017 PMC6920129

[B105] Weng J. Gu S. Wan X. Gao H. Guo T. Su N.. (2008). Isolation and initial characterization of GW5, a major QTL associated with rice grain width and weight. Cell Res. 18, 1199–1209. doi: 10.1038/cr.2008.307 19015668

[B106] Wu Y. Wang Y. Mi X. Shan J. Li X. Xu J.. (2016). The QTL GNP1 encodes GA20ox1, which increases grain number and yield by increasing cytokinin activity in rice panicle meristems. PloS Genet. 12, e1006386. doi: 10.1371/journal.pgen.1006386 27764111 PMC5072697

[B107] Xiao N. Gao Y. Qian H. Gao Q. Wu Y. Zhang D.. (2018). Identification of genes related to cold tolerance and a functional allele that confers cold tolerance. Plant Physiol. 177, 1108–1123. doi: 10.1104/pp.18.00209 29764927 PMC6052991

[B108] Xu Z. Wang H. (2007). LTR_FINDER: an efficient tool for the prediction of full-length LTR retrotransposons. Nucleic Acids Res. 35, W265–W268. doi: 10.1093/nar/gkm286 17485477 PMC1933203

[B109] Xu Y. Wang R. Wang Y. Zhang L. Yao S. (2020). A point mutation in LTT1 enhances cold tolerance at the booting stage in rice. Plant Cell Environ. 43, 992–1007. doi: 10.1111/pce.13717 31922260 PMC7154693

[B110] Xue W. Xing Y. Weng X. Zhao Y. Tang W. Wang L.. (2008). Natural variation in Ghd7 is an important regulator of heading date and yield potential in rice. Nat. Genet. 40, 761–767. doi: 10.1038/ng.143 18454147

[B111] Yan W. Wang P. Chen H. Zhou H. Li Q. Wang C.. (2011). A major QTL, Ghd8, plays pleiotropic roles in regulating grain productivity, plant height, and heading date in rice. Mol. Plant 4, 319–330. doi: 10.1093/mp/ssq070 21148627

[B112] Yang L. Zhao M. Sha G. Sun Q. Gong Q. Yang Q.. (2022). The genome of the rice variety LTH provides insight into its universal susceptibility mechanism to worldwide rice blast fungal strains. Comput. Struct. Biotechnol. J. 20, 1012–1026. doi: 10.1016/j.csbj.2022.01.030 35242291 PMC8866493

[B113] Yano M. Katayose Y. Ashikari M. Yamanouchi U. Monna L. Fuse T.. (2000). Hd1, a major photoperiod sensitivity quantitative trait locus in rice, is closely related to the arabidopsis flowering time gene CONSTANS. Plant Cell 12, 2473–2484. doi: 10.1105/tpc.12.12.2473 11148291 PMC102231

[B114] Yano K. Ookawa T. Aya K. Ochiai Y. Hirasawa T. Ebitani T.. (2014). Isolation of a novel lodging resistance QTL gene involved in strigolactone signaling and its pyramiding with a QTL gene involved in another mechanism. Mol. Plant 6, ssu131. doi: 10.1093/mp/ssu131 25381289

[B115] Yu J. Hu S. Wang J. Wong G. Li S. Liu B.. (2002). A draft sequence of the rice genome (Oryza sativa l. ssp. indica). Science 296, 79–92. 11935017 10.1126/science.1068037

[B116] Yu J. Miao J. Zhang Z. Xiong H. Zhu X. Sun X.. (2018). Alternative splicing of OsLG3b controls grain length and yield in japonica rice. Plant Biotechnol. J. 16, 1667–1678. 29479793 10.1111/pbi.12903 PMC6097128

[B117] Yu J. Xiong H. Zhu X. Zhang H. Li H. Miao J.. (2017). OsLG3 contributing to rice grain length and yield was mined by ho-LAMap. BMC Biol. 15, 28. doi: 10.1186/s12915-017-0365-7 28385155 PMC5383996

[B118] Zhang J. Chen L. Sun S. Kudrna D. Copetti D. Li W.. (2016a). Building two indica rice reference genomes with PacBio long-read and illumina paired-end sequencing data. Sci. Data, 3, 160076. 27622467 10.1038/sdata.2016.76 PMC5020871

[B119] Zhang J. Chen L. Xing F. Kudrna D. Yao W. Copetti D.. (2016b). Extensive sequence divergence between the reference genomes of two elite indica rice varieties zhenshan 97 and minghui 63. Proc. Natl. Acad. Sci. U.S.A. 113, E5163–E5171. 10.1073/pnas.1611012113 PMC5024649 27535938

[B120] Zhang Z. Li J. Pan Y. Li J. Zhou L. Shi H.. (2017). Natural variation in CTB4a enhances rice adaptation to cold habitats. Nat. Commun. 8, 14788. 28332574 10.1038/ncomms14788 PMC5376651

[B121] Zhang H. Wang Y. Deng C. Zhao S. Zhang P. Feng J.. (2022b). High-quality genome assembly of huazhan and tianfeng, the parents of an elite rice hybrid tian-you-hua-zhan. Sci. China Life Sci. 65, 398–411. 34251582 10.1007/s11427-020-1940-9

[B122] Zhang F. Xue H. Dong X. Li M. Zheng X. Li Z.. (2022a). Long-read sequencing of 111 rice genomes reveals significantly larger pan-genomes. Genome Res. 32, 853–863. 35396275 10.1101/gr.276015.121 PMC9104699

[B123] Zhao Q. Feng Q. Lu H. Li Y. Wang A. Tian Q.. (2018b). Pan-genome analysis highlights the extent of genomic variation in cultivated and wild rice. Nat. Genet. 50, 278–284. 29335547 10.1038/s41588-018-0041-z

[B124] Zhao D. Li Q. Zhang C. Zhang C. Yang Q. Pan L.. (2018a). GS9 acts as a transcriptional activator to regulate rice grain shape and appearance quality. Nat. Commun. 9, 1240. 29588443 10.1038/s41467-018-03616-y PMC5869696

[B125] Zhao J. Wang S. Qin J. Sun C. Liu F. (2020). The lipid transfer protein OsLTPL159 is involved in cold tolerance at the early seedling stage in rice. Plant Biotechnol. J. 18, 756–769. 31469486 10.1111/pbi.13243 PMC7004919

[B126] Zhou Z. Yu Z. Huang X. Liu J. Guo Y. Chen L.. (2022). GenomeSyn: a bioinformatics tool for visualizing genome synteny and structural variations. J. Genet. Genomics 49, 1174–1176. doi: 10.1016/j.jgg.2022.03.013 35436609

